# Development and internal validation of a nomogram for early prediction of hospital-acquired ESKAPE colonization or infection in very preterm infants using indicators available within 24 hours

**DOI:** 10.3389/fped.2026.1847533

**Published:** 2026-06-08

**Authors:** Cheng Wen, Jieling Ma, Lijuan Zhang, Liping Yang, Xiaoxue Luo, Ling Yan

**Affiliations:** Department of Pediatrics, The First Affiliated Hospital of Army Medical University, Chongqing, China

**Keywords:** ESKAPE pathogens, hospital-acquired colonization or infection, infection control, nomogram, very preterm infant

## Abstract

**Background:**

ESKAPE pathogens are a major cause of hospital-acquired colonization or infection among very preterm infants in neonatal intensive care units (*N*ICUs). Early identification of high-risk infants can help prioritize infection-control interventions and guide targeted preventive care.

**Methods:**

A single-center retrospective cohort study was conducted among 465 very preterm infants (gestational age, GA ≤ 32 weeks) admitted to the NICU of a tertiary hospital between January 2015 and June 2025. Infants were randomly divided into training (*n* = 325) and internal validation (*n* = 140) cohorts at a 7:3 ratio. Predictors available within 24 h after birth were screened using least absolute shrinkage and selection operator (LASSO) regression. A multivariable logistic regression model was constructed and presented as a nomogram.

**Results:**

Overall, 77 infants (16.56%) developed hospital-acquired ESKAPE colonization or infection, of which 67.5% (52/77) were first identified within 14 days after birth. Among 109 non-duplicate ESKAPE isolates, the predominant pathogens were *Acinetobacter baumannii* (38/109, 34.86%) and *Klebsiella pneumoniae* (31/109, 28.44%); respiratory specimens were the primary source (90/109, 82.57%). Four predictors were retained in the final model: GA at birth, initial invasive mechanical ventilation, vasoactive exposure within the first 24 h, and 5-minute Apgar score. The nomogram showed an area under the receiver operating characteristic curve (AUC) of 0.786 (95% CI: 0.718–0.853) in the training cohort and 0.770 (95% CI: 0.673–0.866) in the internal validation cohort, indicating moderate discrimination. Calibration curves and decision curve analysis demonstrated good agreement between predicted probabilities and observed risks, with net benefit across a wide range of clinically relevant threshold probabilities. An online prediction tool was also developed (https://newborn.shinyapps.io/dynnomapp/).

**Conclusion:**

We developed and internally validated a nomogram for early prediction of hospital-acquired ESKAPE colonization or infection in very preterm infants using routine clinical indicators obtained within 24 h after birth. The model can support early risk stratification and infection control prioritization in the NICU. External validation and prospective implementation studies are required before routine clinical adoption.

## 1 Introduction

ESKAPE pathogens include *Enterococcus faecium (E. faecium)*, *Staphylococcus aureus (S. aureus)*, *Klebsiella pneumoniae (K. pneumoniae)*, *Acinetobacter baumannii (A. baumannii)*, *Pseudomonas aeruginosa (P. aeruginosa)*, and *Enterobacter species* (*Enterobacter* spp.). These pathogens are highly virulent, capable of biofilm formation, and frequently multidrug-resistant (MDR), making them leading causes of healthcare-associated infections worldwide. Several ESKAPE organisms have been designated as priority pathogens by the World Health Organization (WHO) because of their major public health implications (1, 2). In the neonatal intensive care unit (NICU), environmental contamination levels have been reported to be as high as 93.9%, with nearly one-quarter of isolates belonging to ESKAPE pathogens, 90% of which are MDR (3). Such heavy contamination creates a persistent reservoir for cross-transmission. Very preterm infants with a gestational age (GA) ≤32 weeks are especially vulnerable to hospital-acquired ESKAPE colonization or infection due to immature immune function and frequent exposure to invasive medical procedures (4). Previous studies reported that up to 79% of infants are colonized by at least one ESKAPE pathogen during NICU stay, and 44% of colonization events occur within 24 h after birth, with *K. pneumoniae* and *Enterobacter* spp. being the dominant colonizing strains (5). During outbreaks of extended-spectrum beta-lactamase (ESBL)-producing Enterobacteriaceae in NICUs, *Klebsiella* species are often involved and associated with high mortality in affected infants (6).

Very preterm infants represent a high-risk group for hospital-acquired ESKAPE events (3, 7–9). In an outbreak of *A. baumannii*, infection occurred only in extremely low birth weight (ELBW) infants with GA ≤ 26 weeks and postnatal age ≤7 days, suggesting a narrow and critical window of susceptibility related to developmental immaturity (10). The combined effects of host vulnerability and iatrogenic exposures may further increase the risk of ESKAPE colonization or infection (11). Preterm infants are exposed to frequent clinical procedures during the early postnatal period, particularly suctioning and skin-breaking procedures, with stress exposure and procedural burden being especially high during the first days and weeks after birth (12, 13). These findings underscore a major challenge for infection prevention and control: nurses must not only maintain the physiological stability of these infants, but also perform essential procedures that may inadvertently increase exposure risk.

Current infection control strategies in NICUs are largely reactive: interventions are usually started only after infection is confirmed. However, conventional microbial culture is slow, and by the time results become available, the infant may already be clinically unstable, and the optimal window for intervention may have passed (14, 15). Early detection of ESKAPE colonization is therefore critical, as colonization precedes infection and serves as a major source of transmission (15, 16). A study by Peng et al. among hospitalized children demonstrated that preterm birth/low birth weight (LBW) was an independent risk factor for ESKAPE bloodstream infection (OR = 2.98), suggesting that early clinical data may contain useful risk signals (17).

Although several prediction models for neonatal MDR infections have been published, most focus on the general neonatal population and do not fully address the unique vulnerability of very preterm infants or the specific epidemiology of ESKAPE pathogens (18, 19). In addition, many models use variables available beyond the first 24 h, limiting their utility for early bedside decision-making (18). Prevention is further complicated by the biological characteristics of ESKAPE pathogens. *K. pneumoniae* and *P. aeruginosa* are well-recognized biofilm-forming pathogens, and biofilm formation can reduce antibiotic susceptibility and protect bacteria from host immune clearance, thereby favoring persistent colonization (20, 21). *A. baumannii* has been identified as an important environmental contaminant and pathogen in NICU settings and may contribute to sustained environmental reservoirs and hospital cross-transmission (3). Together, these characteristics reduce the effectiveness of conventional disinfection and isolation strategies.

Accordingly, this study aimed to develop and internally validate a nomogram using clinical variables available within 24 h after birth to predict hospital-acquired ESKAPE colonization or infection in very preterm infants. This model is intended to enable early risk stratification and help optimize infection control resource allocation in the NICU.

## 2 Materials and methods

### 2.1 Study design and population

This was a single-center retrospective cohort study of very preterm infants (GA ≤ 32 weeks) admitted to the NICU of a tertiary hospital between January 2015 and June 2025. The study was approved by the Ethics Committee of the First Affiliated Hospital of Army Medical University [Approval No. (B)KY2026092]. Informed consent was waived due to the retrospective nature of the study. All procedures were performed in accordance with the Declaration of Helsinki.

### 2.2 Inclusion and exclusion criteria

Inclusion criteria were as follows: (1) admission within 24 h after birth; (2) hospital stay longer than 48 h; (3) at least one microbiological culture obtained during hospitalization, including blood, sputum, catheter-tip, or other clinical specimens; and (4) complete clinical data without missing values for all variables included in the analysis. The requirement for hospitalization longer than 48 h was used to ensure an adequate time window for evaluating early exposures and subsequent hospital-acquired events (22).

Exclusion criteria were as follows: (1) detection of ESKAPE pathogens in specimens obtained within the first 48 h after admission, suggesting community-acquired or pre-admission colonization or infection; (2) presence of severe fatal congenital malformations or complex chromosomal abnormalities; and (3) incomplete clinical data.

**Figure 1:**
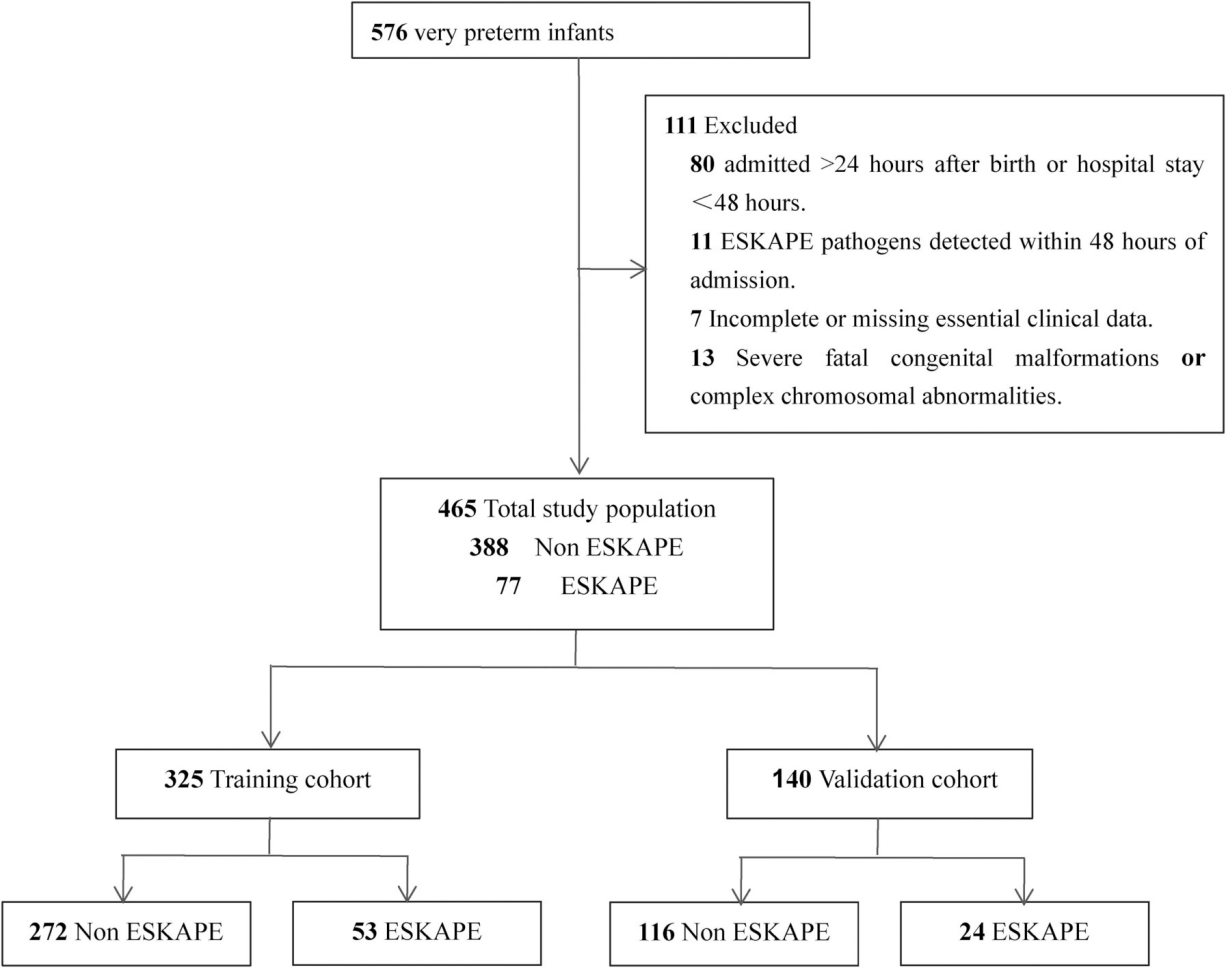
Flowchart of patient selection.

A total of 465 infants were included in the final analysis. They were randomly divided into a training cohort (*n* = 325) and an internal validation cohort (*n* = 140) at a ratio of 7:3. The flowchart of participant selection is shown in Figure 1.

### 2.3 Outcome definition

The primary outcome was hospital-acquired ESKAPE colonization or infection, defined as the first isolation of ESKAPE pathogens from any clinical specimen (e.g., blood, sputum, endotracheal aspirate, catheter tip, urine, or cerebrospinal fluid) obtained more than 48 h after admission. Colonization was defined as a positive culture result without corresponding clinical manifestations, i.e., no infection-related symptoms, signs, or imaging abnormalities; infection was defined as a positive culture result accompanied by compatible clinical signs and symptoms (e.g., temperature instability, apnea, bradycardia, feeding intolerance). To avoid duplicate counting, multiple isolations of the same strain from the same site in the same infant were counted as a single event, whereas isolations of different strains or from different sites were recorded separately for descriptive microbiological analyses, but not counted as additional primary outcome events.

In this study, colonization and infection were combined as a composite endpoint from an infection-control perspective. In the context of ESKAPE pathogens prevention and control, both states often involve the same pathogen group, similar transmission pathways, and overlapping preventive strategies, including contact isolation, environmental cleaning and disinfection, and reinforced hand hygiene. In very preterm infants, especially when respiratory and other non-sterile-site specimens predominate, the distinction between colonization and infection can be clinically challenging because neonatal signs are often nonspecific and microbiological criteria may have limited specificity (9, 23). In addition, colonization frequently precedes infection and may serve as an important reservoir for onward transmission in the NICU (16, 24). Therefore, consistent with the goal of developing an early infection-control risk warning tool, rather than a treatment-guidance model, combining these two outcomes was considered appropriate to more comprehensively identify infants requiring intensified preventive interventions.

### 2.4 Data collection

Clinical and microbiological data were retrospectively extracted from the hospital's electronic medical record system using a standardized electronic case report form. Candidate predictors were selected based on a literature review and clinical judgment, including (1) demographic characteristics: sex, GA recorded in days, BW; (2) perinatal factors: maternal age, gestational diabetes mellitus, gestational hypertension, duration of premature rupture of membranes (PROM), chorioamnionitis, antenatal corticosteroid therapy, and antenatal antibiotic exposure; (3) neonatal condition at birth: 1-minute and 5-minute Apgar scores; (4) interventions within 24 h after birth: initial invasive mechanical ventilation (IMV), vasoactive exposure, antibiotic exposure, and placement of a peripherally inserted central catheter (PICC). All records were reviewed independently by two investigators (Jieling Ma and Lijuan Zhang, both co-authors of this study) and cross-checked to ensure data quality. All data were de-identified before analysis. The electronic data were stored on a secure, password-protected hospital server with access limited to the research team. In accordance with institutional and national data protection regulations, the data will be retained for a defined period and then securely deleted.

Definitions of key interventions were as follows: Antenatal magnesium sulfate therapy was defined as maternal intravenous administration of magnesium sulfate for fetal neuroprotection. Antenatal corticosteroid therapy was defined as maternal corticosteroid administration to promote fetal lung maturation, typically dexamethasone 6 mg every 12 h for four doses (25). Antenatal maternal antibiotic exposure was defined as the administration of any systemic antibiotic to the mother during labor. Vasoactive exposure within the first 24 h after birth was defined as the continuous intravenous infusion of dopamine, dobutamine, epinephrine, or norepinephrine. All interventions were coded as binary variables (yes/no), indicating whether the intervention was administered, rather than capturing cumulative dose, treatment duration, or completion of a full course.

### 2.5 Statistical methods

Data analysis was performed using R software (version 4.3.0). The normality of continuous data was tested using the Shapiro–Wilk test. Normally distributed data were presented as mean ± standard deviation, and the independent-sample *t*-test was used for between-group comparisons. Non-normally distributed data were presented as median (interquartile range), and the Mann–Whitney *U* test was used for between-group comparisons. Categorical data were presented as *n* (%), and the *χ*^2^ test or Fisher's exact test was used for between-group comparisons. All hypothesis tests were two-sided, and *P* < 0.05 was considered statistically significant. The total sample was randomly divided into a training cohort (*n* = 325) and an internal validation cohort (*n* = 140) at a 7:3 ratio. The training cohort was used for model development, whereas the internal validation cohort was used to assess model performance in patients not included in the training cohort. In the training cohort, continuous predictors were standardized as *z*-scores, and categorical variables were coded as binary variables (0/1). Feature selection was performed using least absolute shrinkage and selection operator (LASSO) regression, with the optimal penalty parameter *λ* determined by 10-fold cross-validation. Variables with non-zero coefficients were subsequently included in a multivariable logistic regression model to construct the final nomogram. The final model included 53 outcome events and four predictors, yielding an EPV of 13.25. To reduce optimism and assess the robustness of model performance, internal validation was performed using 1,000 bootstrap resamples. Discrimination was evaluated using the area under the receiver operating characteristic curve (AUC), calibration was assessed using calibration plots, and clinical utility was evaluated using decision curve analysis (DCA). The optimal cutoff value was determined using the Youden index from the receiver operating characteristic (ROC) curve in the training cohort.

## 3 Results

### 3.1 Study population and etiological characteristics

**Figure 2:**
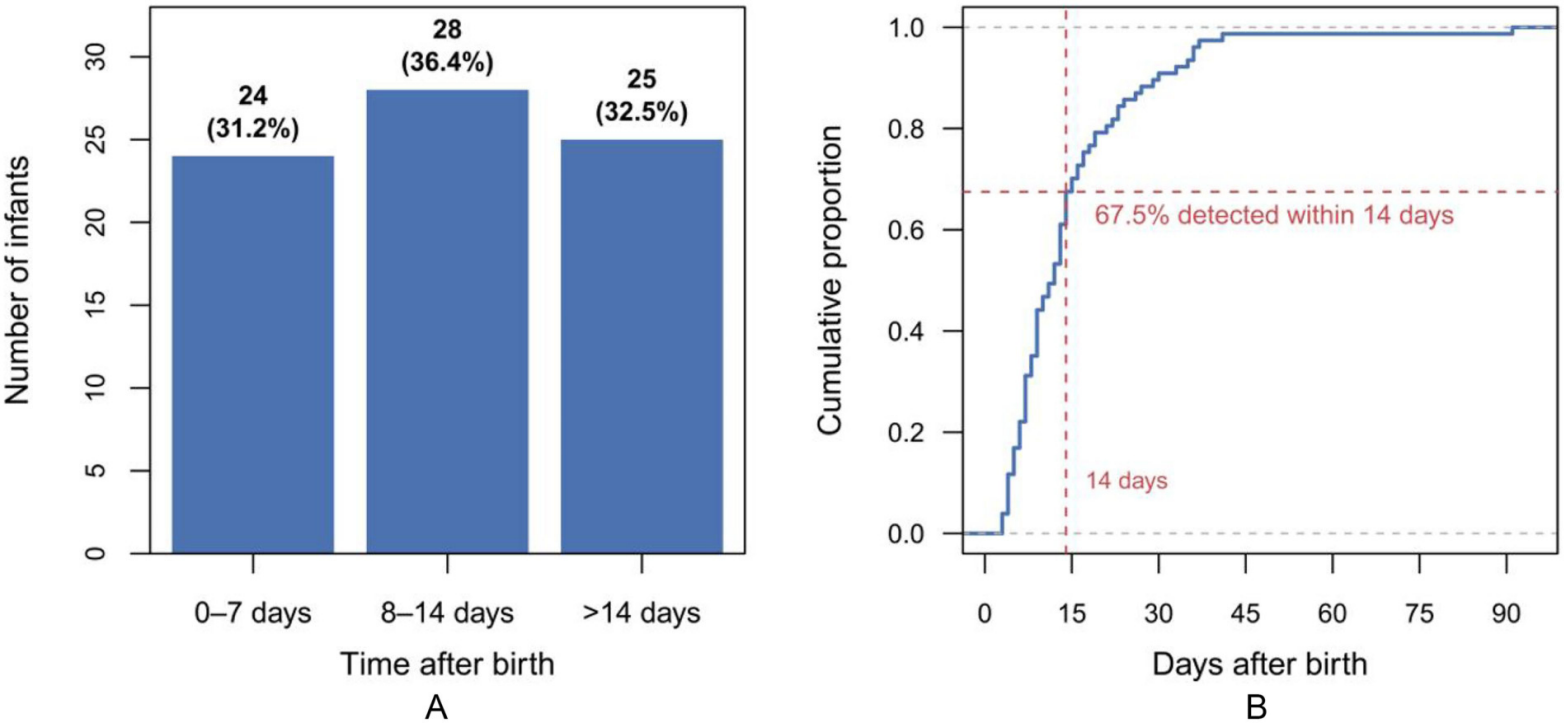
Time to first detection of ESKAPE pathogens in very preterm infants. **(A)** Bar chart showing the distribution of the first positive culture by time intervals: 0–7 days (*n* = 24, 31.2%), 8–14 days (*n* = 28, 36.4%), and >14 days after birth (*n* = 25, 32.5%). **(B)** Cumulative incidence curve showing that 67.5% of hospital-acquired ESKAPE colonization or infection events were identified within the first 14 days after birth.

Of the 465 very preterm infants enrolled, 77 (16.6%) developed hospital-acquired ESKAPE colonization or infection, including 53 in the training cohort and 24 in the validation cohort. As shown in Figure 2, 67.5% (52/77) of these events were first identified within the first 14 days after birth, indicating that this period may represent a high-risk window for ESKAPE acquisition in this population.

**Figure 3:**
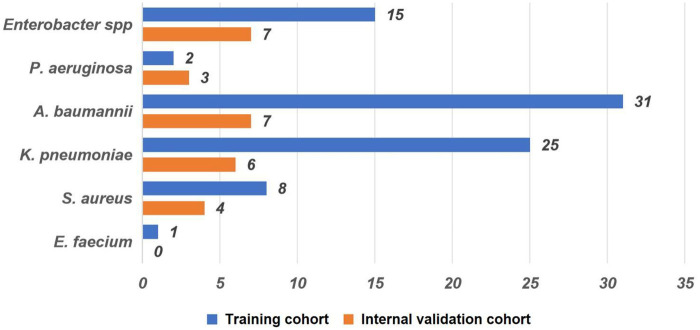
Distribution of ESKAPE pathogens in the training and internal validation cohorts. *A. baumannii*, *Acinetobacter baumannii*; *K. pneumoniae*, *Klebsiella pneumoniae*; *P. aeruginosa*, *Pseudomonas aeruginosa*; *E. faecium*, *Enterococcus faecium*; *S. aureus*, *Staphylococcus aureus*; *Enterobacter* spp., *Enterobacter* species.

**Figure 4:**
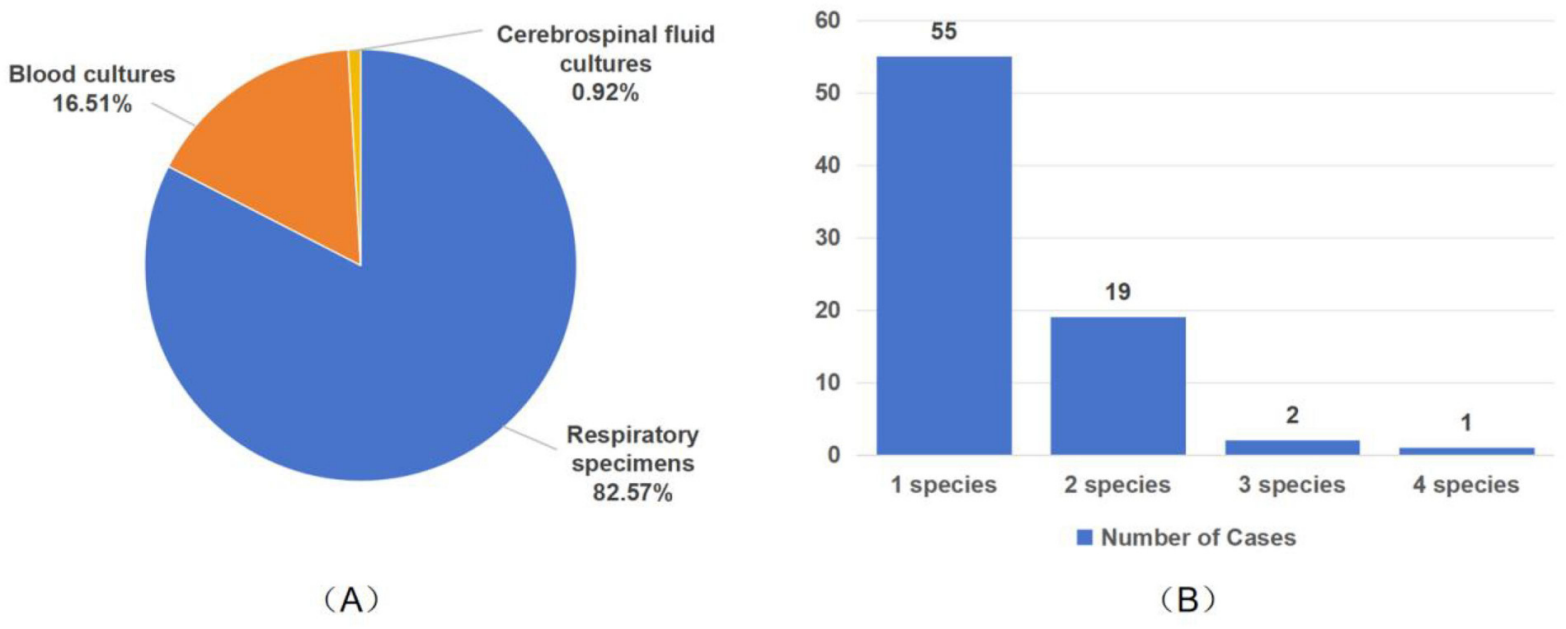
Clinical features of hospital-acquired ESKAPE colonization or infection in 77 very preterm infants. **(A)** Distribution of clinical specimen sources; **(B)** distribution of the number of ESKAPE pathogen species isolated per infant.

In this study, the distribution of ESKAPE pathogens demonstrated a distinct distribution across species. For the primary outcome analysis, each infant was counted only once, based on the first qualifying event of hospital-acquired ESKAPE colonization or infection. For descriptive microbiological analyses, non-duplicate isolates from different strains or sites were additionally summarized: *A. baumannii* was the most common (31 cases in the training cohort, 7 cases in the validation cohort), while *E. faecium* was the least common (1 case in the training cohort, 0 cases in the validation cohort), with a generally consistent pathogen distribution between the training and internal validation cohorts (Figure 3). Regarding specimen sources (Figure 4A), respiratory tract specimens accounted for the highest proportion (82.57%), followed by blood cultures (16.51%) and cerebrospinal fluid cultures (0.92%). In terms of infection type, a single pathogen was detected in 55 cases (71.43%), two pathogens (mixed or sequential) were detected in 19 cases (24.68%), and three or more pathogens were detected in 3 cases (3.90%), indicating that hospital-acquired ESKAPE colonization or infection in very preterm infants was predominantly caused by single strains (Figure 4B).

### 3.2 Baseline characteristics

The training and internal validation cohorts were generally balanced and comparable in most baseline characteristics with significant differences observed only in absolute neutrophil count (*P* = 0.033) and base excess (*P* = 0.043), suggesting that the cohort allocation was overall balanced with favorable internal comparability. Among the 325 infants in the training cohort, 53 were in the ESKAPE group and 272 in the non-ESKAPE group. Baseline characteristics are summarized in Table 1. Notably, the between-group *P* values were not adjusted for multiple testing and should be regarded as exploratory results.

**Table 1:** Patient demographics and baseline characteristics.

Variables	Training cohort (*n* = 325)	Internal validation Cohort (*n* = 140)	*P* ^1^	Non-ESKAPE (*n* = 272)	ESKAPE (*n* = 53)	*P* ^2^
GA (days)	212 (204, 219)	214 (203, 219)	0.519	213 (206, 219)	204 (198, 215)	<0.001
BW (g)	1,350 (1,115, 1,550)	1,350 (1,153, 1,565)	0.736	1,388 (1,130, 1,570)	1,220 (1,080, 1,380)	0.005
Male (yes, %)	172 (52.9%)	72 (51.4%)	0.767	137 (50.4%)	35 (66.0%)	0.037
Multiple gestations (yes, %)	95 (29.2%)	41 (29.3%)	0.990	76 (27.9%)	19 (35.8%)	0.247
Delivery room intubation (yes, %)	62 (19.1%)	35 (25.0%)	0.149	41 (15.1%)	21 (39.6%)	<0.001
1-minute Apgar score	9.00 (8.00, 10.00)	9.00 (7.00, 10.00)	0.740	9.00 (8.00, 10.00)	8.00 (5.00, 9.00)	<0.001
5-minute Apgar score	10.00 (9.00, 10.00)	10.00 (9.00, 10.00)	0.346	10.00 (9.00, 10.00)	9.00 (8.00, 10.00)	<0.001
Hospitalization in spring or winter (yes, %)	176 (54.2%)	85 (60.7%)	0.191	136 (50.0%)	40 (75.5%)	<0.001
Admission hypothermia (yes, %)	69 (21.2%)	27 (19.3%)	0.625	57 (21.0%)	12 (22.6%)	0.784
Cesarean delivery (yes, %)	187 (57.5%)	77 (55.0%)	0.742	163 (59.9%)	24 (45.3%)	0.05
Advanced maternal age pregnancy (yes, %)	64 (19.7%)	27 (19.3%)	0.919	53 (19.5%)	11 (20.8%)	0.832
Gestational diabetes mellitus (yes, %)	87 (26.8%)	39 (27.9%)	0.809	78 (28.7%)	9 (17.0%)	0.079
Hypertensive disorders of pregnancy (yes, %)	69 (21.2%)	37 (26.4%)	0.220	59 (21.7%)	10 (18.9%)	0.646
PROM (h)	0 (0, 24)	0 (0, 9)	0.154	0 (0, 24)	0 (0, 24)	0.909
Antenatal maternal fever (yes, %)	16 (4.9%)	7 (5.0%)	0.972	12 (4.4%)	4 (7.5%)	0.308
Antenatal maternal antibiotic exposure (yes, %)	182 (56.0%)	79 (56.4%)	0.932	152 (55.9%)	30 (56.6%)	0.923
Chorioamnionitis (yes, %)	62 (19.1%)	27 (19.3%)	0.958	54 (19.9%)	8 (15.1%)	0.420
Placental abnormalities (yes, %)	100 (30.8%)	38 (27.1%)	0.432	82 (30.1%)	18 (34.0%)	0.582
Antenatal corticosteroid therapy (yes, %)	214 (65.8%)	92 (65.7%)	0.978	176 (64.7%)	38 (71.7%)	0.326
Antenatal magnesium sulfate therapy (yes, %)	179 (55.1%)	80 (57.1%)	0.681	144 (52.9%)	35 (66.0%)	0.080
Initial IMV (yes, %)	149 (45.8%)	68 (48.6%)	0.589	110 (40.4%)	39 (73.6%)	<0.001
Pulmonary surfactant therapy (yes, %)	213 (65.5%)	103 (73.6%)	0.089	170 (62.5%)	43 (81.1%)	0.009
PICC/UVC/UAC (yes, %)	190 (58.5%)	85 (60.7%)	0.650	160 (58.8%)	30 (56.6%)	0.764
Antibiotic exposure within first 24 h (yes, %)	251 (77.2%)	117 (83.6%)	0.123	203 (74.6%)	48 (90.6%)	0.011
Vasoactive exposure within first 24 h (yes, %)	97 (29.8%)	38 (27.1%)	0.556	66 (24.3%)	31 (58.5%)	<0.001
FIO_2_ (%)	40 (34, 40)	40 (30, 40)	0.538	40 (30, 40)	40 (40, 50)	<0.001
pH	7.33 (7.27, 7.39)	7.31 (7.26, 7.38)	0.285	7.33 (7.26, 7.39)	7.32 (7.27, 7.38)	0.901
Base excess (mmol/L)	−4.5 (−6.3, −2.9)	−5.3 (−7.4, −3.0)	0.043	−4.3 (−6.2, −2.7)	−5.4 (−10.1, −3.6)	0.011
Hemoglobin (g/L)	162 (143, 181)	161 (143, 179)	0.738	164 (144, 182)	155 (135, 175)	0.053
Platelet count (×10^9^/L)	221 (174, 266)	227 (174, 260)	0.966	222 (180, 267)	204 (150, 254)	0.127
Absolute neutrophil counts (×10^9^/L)	3.5 (2.2, 5.8)	4.4 (2.5, 6.9)	0.033	3.4 (2.1, 5.4)	3.8 (2.5, 7.6)	0.092

Data are presented as *n* (%) or median (interquartile range). Exploratory *P*-values should be interpreted with caution due to multiple testing. *P*^1^ is for the comparison between the training and internal validation cohorts; *P*^2^ is for the comparison between the non-ESKAPE and ESKAPE groups within the training cohort. GA, gestational age; BW, birth weight; PROM, premature rupture of membranes; IMV, invasive mechanical ventilation; PICC, peripherally inserted central catheter; UVC, umbilical venous catheter; UAC, umbilical arterial catheter; FiO_2_, fraction of inspired oxygen. GA was recorded and analyzed in days to improve precision; GA ≤ 32 weeks was used only as the eligibility criterion.

In the training cohort, 53 of 325 infants (16.3%) developed ESKAPE colonization or infection. Compared with the non-ESKAPE group, infants in the ESKAPE group had lower GA [204 (198, 215) vs. 213 (206, 219) days, *P* < 0.001] and BW [1,220 (1,080, 1,380) vs. 1,388 (1,130, 1,570) g, *P* = 0.005], were more likely to be male and hospitalized in winter or spring, and had lower 1- and 5-minute Apgar scores. Most perinatal factors were similar between groups, including gestational diabetes mellitus, hypertensive disorders of pregnancy, PROM, antenatal maternal fever, chorioamnionitis, placental abnormalities, antenatal corticosteroid therapy and magnesium sulfate therapy, multiple gestations, and central catheter placement, although cesarean delivery showed a borderline difference (45.3% vs. 59.9%, *P* = 0.05).

Infants in the ESKAPE group more frequently required delivery room intubation, IMV, pulmonary surfactant therapy, early antibiotic exposure, and vasoactive exposure within 24 h after birth. They also required a higher fraction of inspired oxygen (FiO_2_) and had more severe metabolic acidosis, reflected by lower base excess. Laboratory parameters, including pH, hemoglobin, platelet count, and absolute neutrophil counts, did not differ significantly between the groups (*P* > 0.05).

### 3.3 Predictor selection using LASSO regression

**Figure 5:**
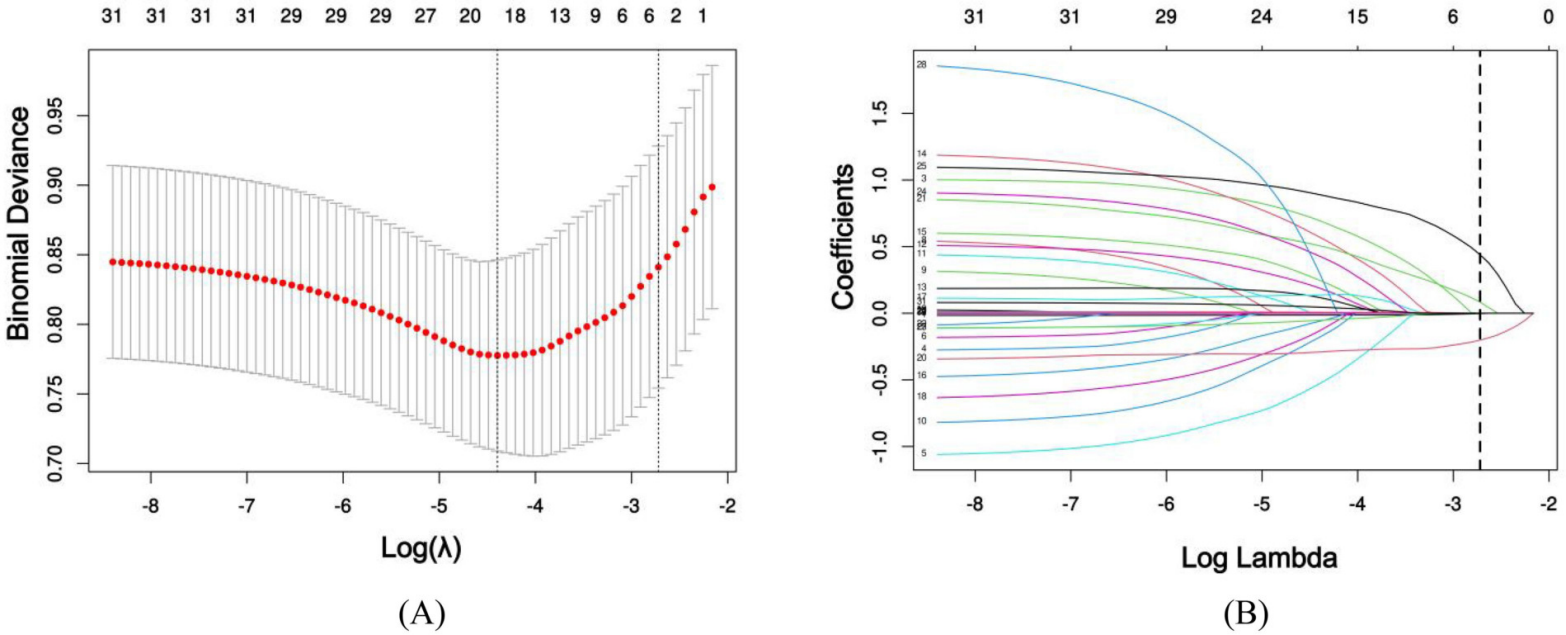
Features were selected using LASSO binary logistic regression. **(A)** Ten-fold cross-validation using the minimum cross-validation criterion was performed to identify the optimal *λ*. **(B)** The trajectory of coefficients for the four features was plotted against a range of log(*λ*) values.

**Figure 6:**
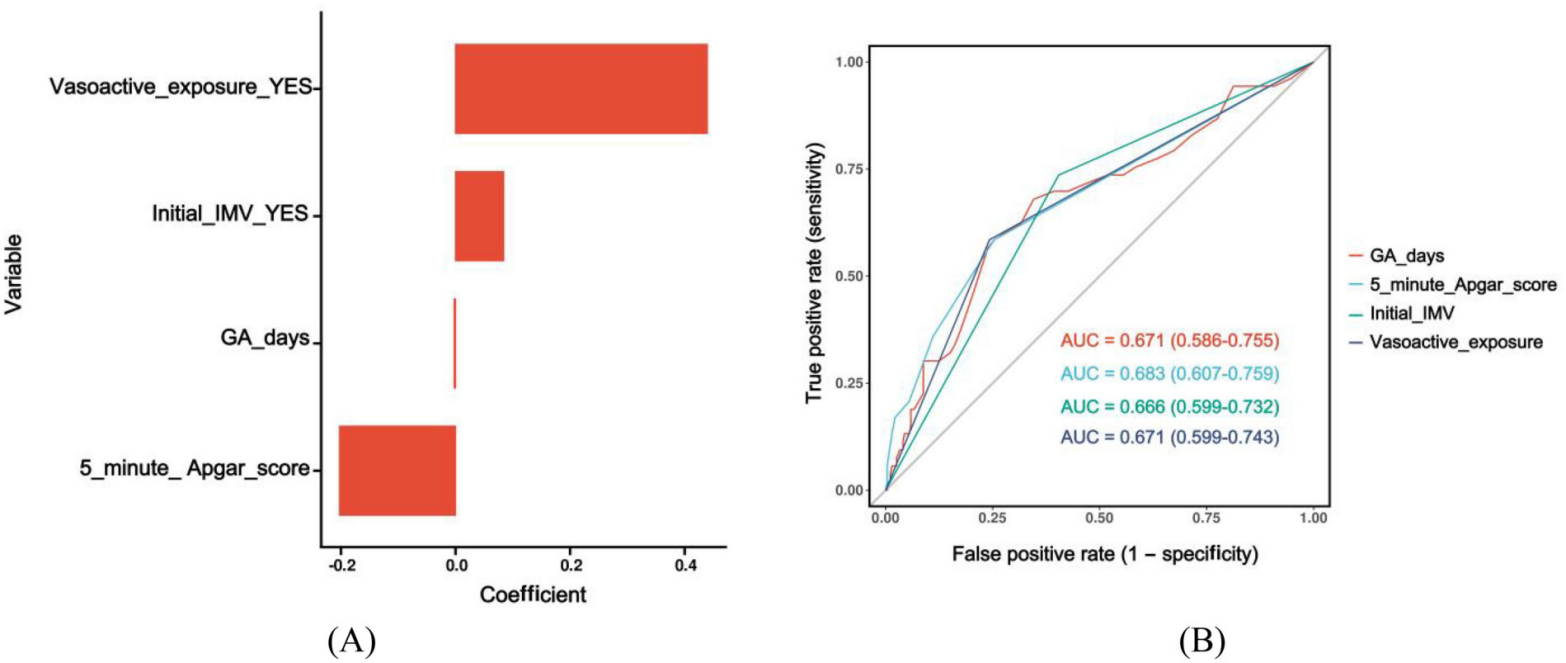
Feature selection with LASSO regression. **(A)** Coefficients of the four features selected by the LASSO algorithm. A positive coefficient indicates that the presence of the factor is associated with an increased risk of ESKAPE colonization or infection. Negative coefficients for GA and 5-minute Apgar score indicate that lower GA or lower Apgar score is associated with increased risk. **(B)** ROC curves evaluating the performance of the prediction model.

To comprehensively evaluate predictive performance and avoid information loss, all baseline variables presented in Table 1 were entered as candidate predictors in the LASSO regression analysis without prior selection. This approach ensured that no potentially relevant variables were excluded. The LASSO algorithm (Figure 5) identified four predictors with non-zero coefficients: initial IMV, vasoactive exposure within the first 24 h, GA at birth, and 5-minute Apgar score (Figure 6A). Figure 6B shows the ROC curves and corresponding AUC values (0.666, 0.671, 0.671, and 0.683) for the four predictive models, indicating modest discriminatory ability.

### 3.4 Construction of the nomogram model

**Figure 7:**
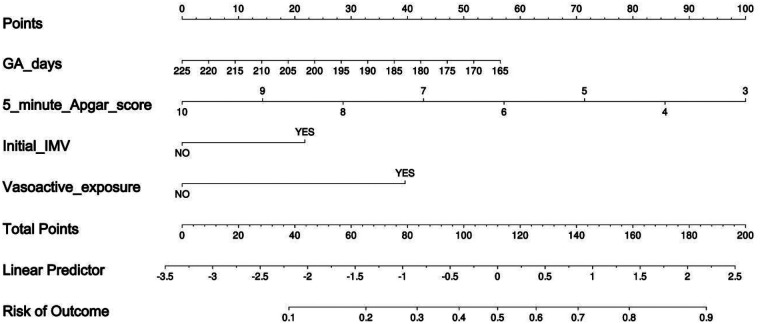
Nomogram for predicting hospital-acquired ESKAPE colonization or infection in very preterm infants.

A nomogram was developed based on four predictors identified by multivariable logistic regression, as shown in Figure 7. The final regression model was expressed as ln[P/(1-P)] = 7.196–0.028 × (GA)−0.424 × (5-minute Apgar score) + 0.646 × (initial IMV) + 1.174 × (vasoactive exposure within the first 24 h). A higher total score indicated a higher probability of ESKAPE colonization or infection.

**Figure 8:**
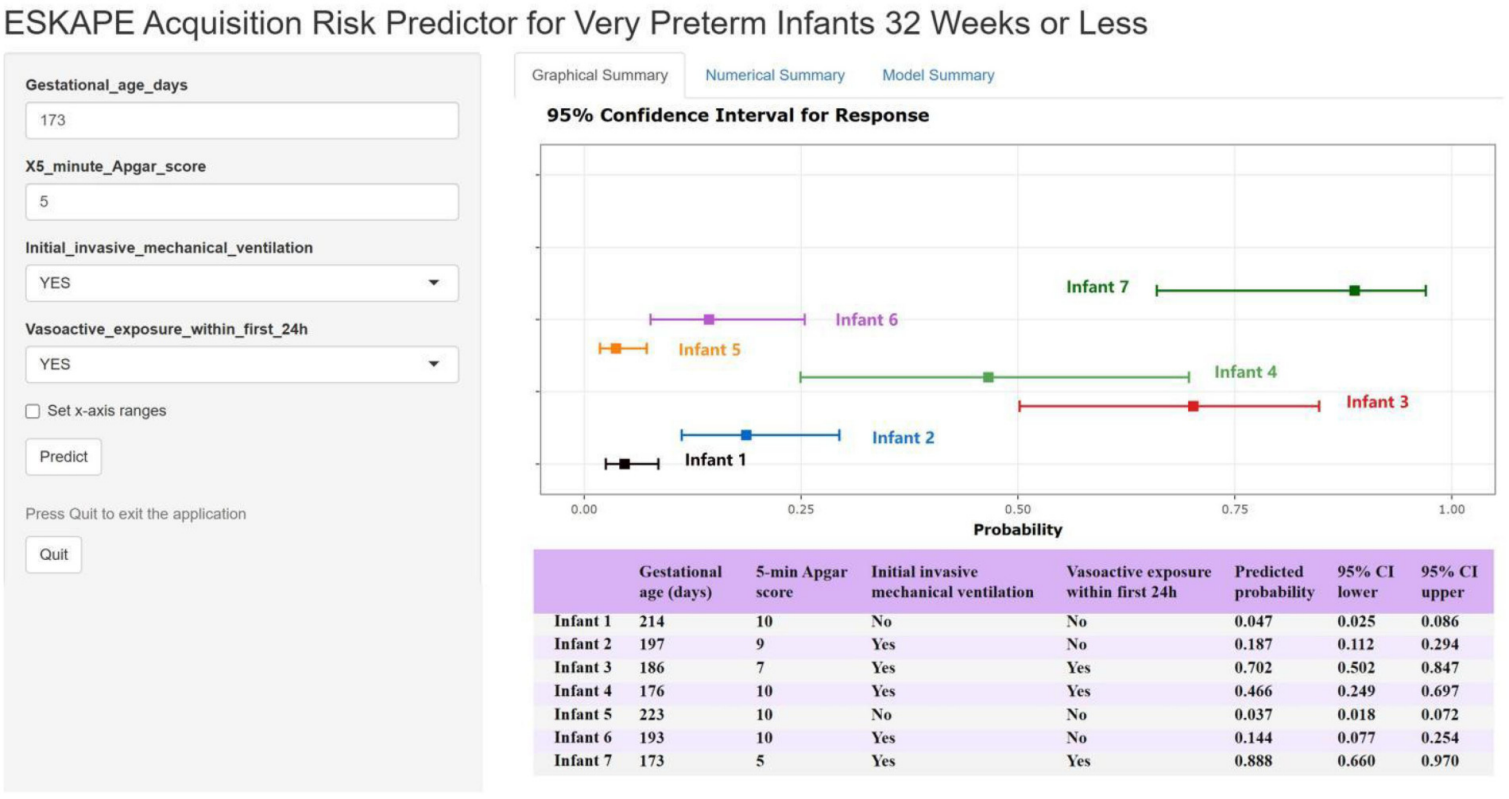
Online nomogram for ESKAPE colonization or infection prediction in very preterm infants (https://newborn.shinyapps.io/dynnomapp/). Left panel: input variables; right panel: predicted probability (95% CI). Example variable values and corresponding predicted probabilities are shown in the table.

To illustrate its clinical application, a representative case was evaluated. An infant with a GA of 205 days, a 5-minute Apgar score of 10, and receipt of both initial IMV and vasoactive agents had corresponding nomogram scores of approximately 18 points (GA), 0 points (5-minute Apgar score), 22 points (initial IMV), and 40 points (vasoactive exposure), yielding a total score of approximately 80 points and a predicted risk of approximately 28% for ESKAPE colonization or infection. A web-based calculator was also developed (https://newborn.shinyapps.io/dynnomapp/), enabling real-time estimation of individualized risk after entering clinical parameters (Figure 8).

### 3.5 Validation of the nomogram model

**Figure 9:**
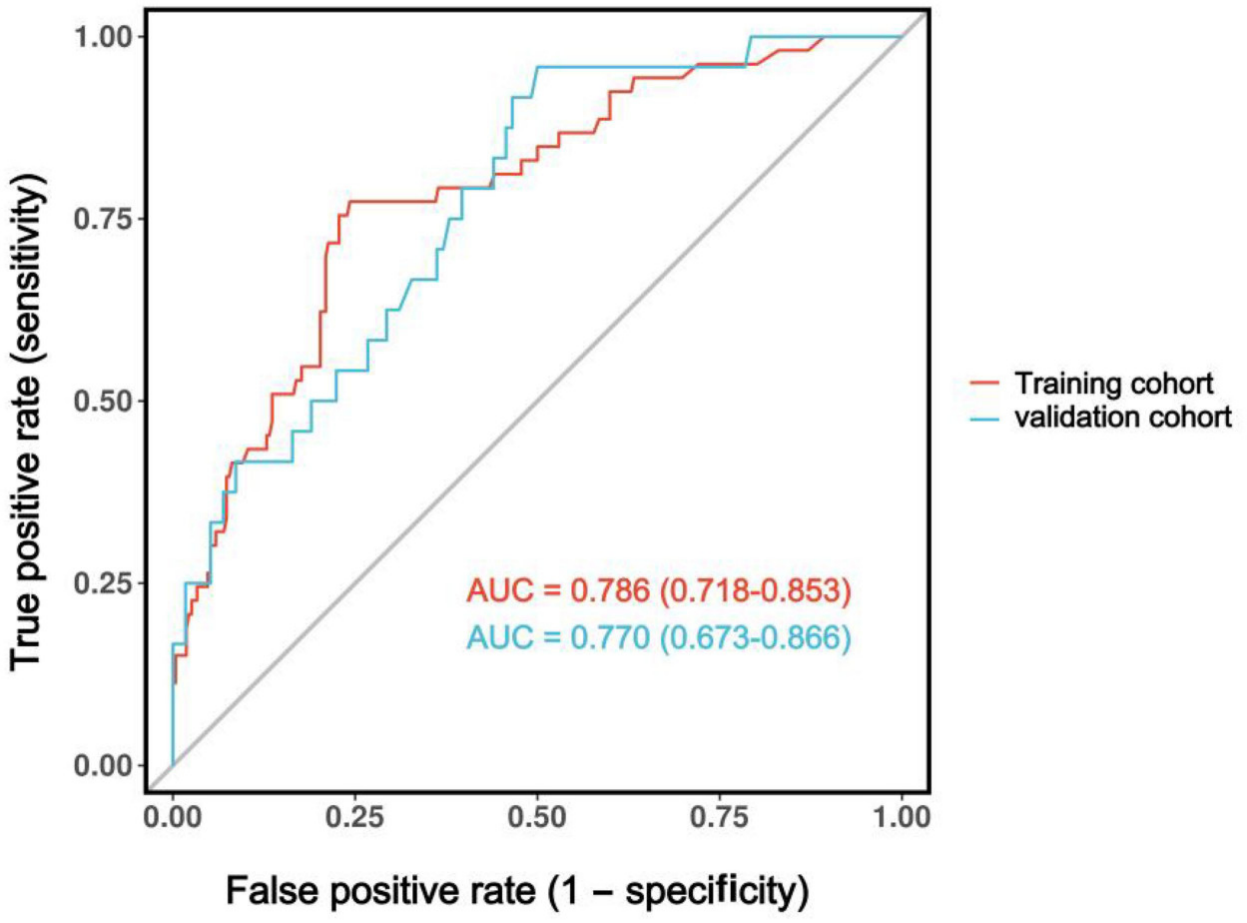
ROC curves for predicting ESKAPE colonization or infection in very preterm infants in the training and internal validation cohorts.

The predictive model demonstrated moderate discrimination, with an AUC of 0.786 (95% CI: 0.718–0.853) in the training cohort and 0.770 (95% CI: 0.673–0.866) in the internal validation cohort (Figure 9). The optimal cutoff probability was 0.157, corresponding to a sensitivity of 77.4%, a specificity of 75.7%, a Youden index of 0.531 (Supplementary Figure S1), and a total nomogram score of 61.2 (on a 0–200 scale).

**Figure 10:**
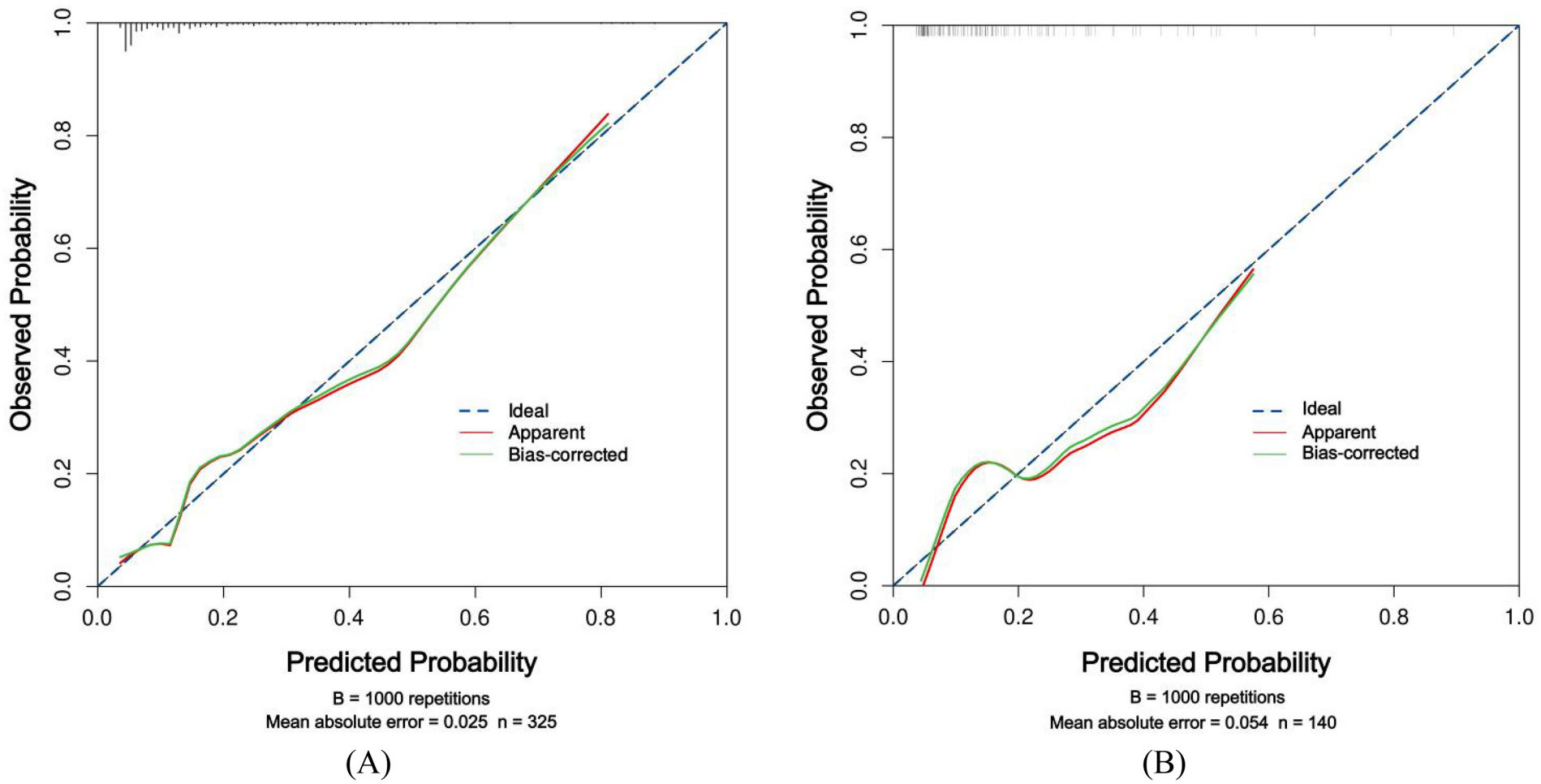
Calibration curves depicting observed vs. predicted probabilities of ESKAPE colonization or infection in the training **(A)** and internal validation **(B)** cohorts. The *x*-axis corresponds to the nomogram-predicted probability, while the *y*-axis indicates the actual observed frequency.

**Figure 11:**
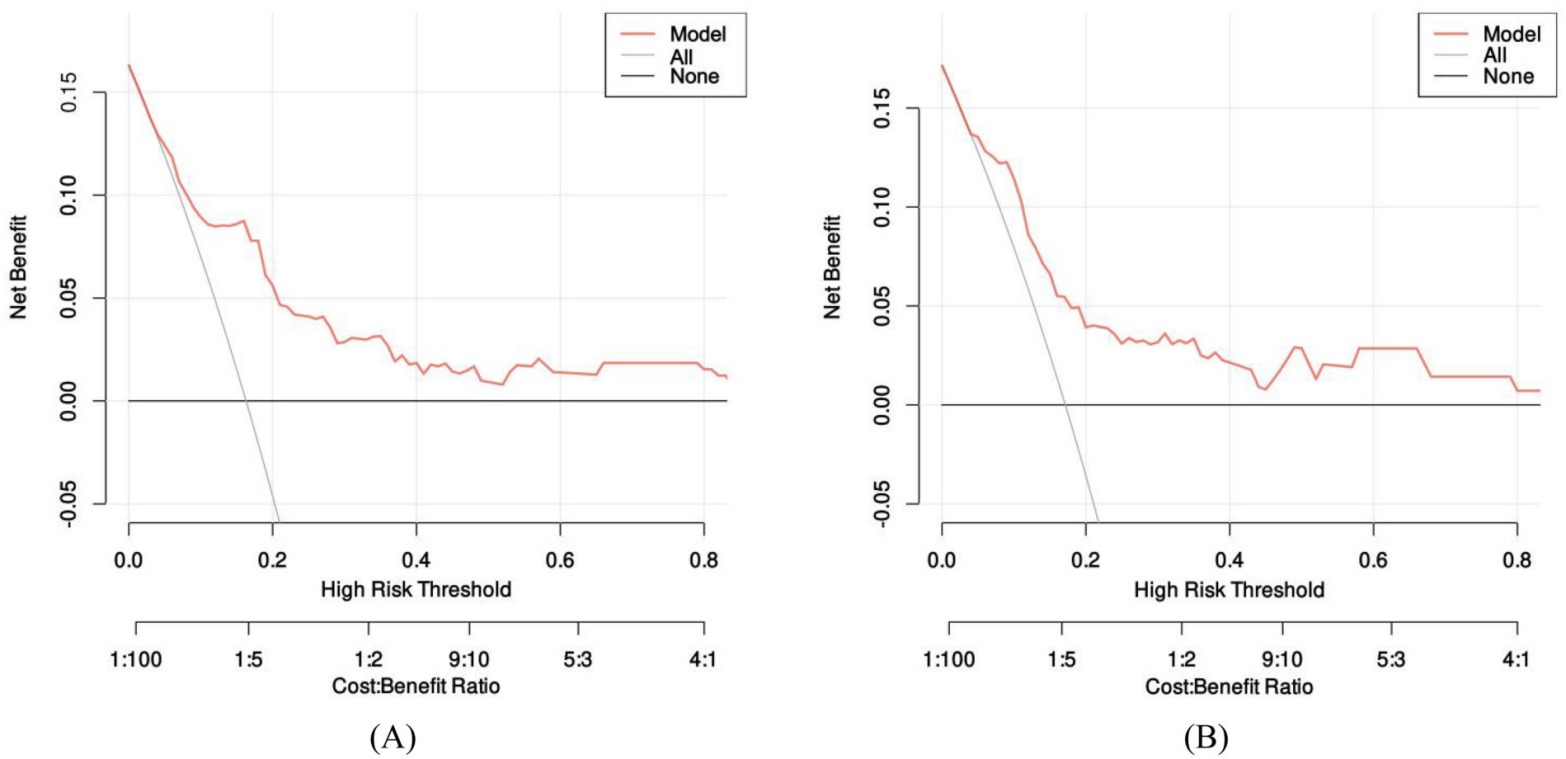
DCA curves for ESKAPE prediction in very preterm infants: **(A)** training cohort; **(B)** validation cohort.

Bootstrap validation with 1,000 resamples demonstrated good calibration in both cohorts, as indicated by the close agreement between the calibration curves and the ideal reference line (Figure 10). Decision curve analysis demonstrated that the model provided a net clinical benefit across a wide range of threshold probabilities (0.1–0.8) in both cohorts, compared with the “treat-all” and “treat-none” strategies (Figure 11).

## 4 Discussion

To our knowledge, this study developed and internally validated one of the first nomograms for predicting hospital-acquired ESKAPE colonization or infection in very preterm infants (GA ≤ 32 weeks) using clinical indicators available within 24 h after birth. The four predictors identified by LASSO (GA, 5-minute Apgar score, initial IMV, vasoactive exposure) reflect the combined effects of physiological immaturity, perinatal stress, and invasive interventions on early infection risk. Importantly, the model is intended for early risk stratification rather than causal inference. By using routinely available early admission variables, it may help NICU teams identify infants requiring intensified infection control measures during the most vulnerable hospitalization period and support earlier, risk-directed preventive care (3).

A key methodological feature is the use of a composite outcome (colonization plus infection). This choice was driven by infection control priorities: in the NICU, colonization and infection share similar pathogens, transmission routes, and preventive strategies, and colonized infants act as a key reservoir for transmission (24). Thus, combining outcomes is reasonable for an early warning tool guiding isolation, cleaning, and enhanced prevention. We acknowledge that colonization and infection differ in clinical management, and this composite endpoint may reduce specificity for treatment decisions. The model should therefore be viewed primarily as an infection control warning tool rather than a treatment-guidance model.

In this cohort, the overall incidence of hospital-acquired ESKAPE colonization or infection was 16.56% (77/465), with 67.5% of events detected within 14 days after birth. This suggests that the first two postnatal weeks may represent a critical window for pathogen acquisition in very preterm infants, during which intensified surveillance and preventive care may be particularly valuable. The pathogen spectrum was dominated by *A. baumannii* (34.87%) and *K. pneumoniae* (28.44%), consistent with neonatal and NICU studies showing that Gram-negative organisms are major pathogens in hospital-acquired neonatal infections (26). In our cohort, respiratory specimens accounted for 82.57% of positive specimens, indicating that the respiratory tract may be a major acquisition site in this population (27, 28). These findings support the clinical relevance of an early risk-stratification model and underscore the importance of respiratory care in infection prevention and control among very preterm infants (29–31).

Initial IMV was retained (OR = 1.91), indicating that early invasive respiratory support marks higher illness severity and infection risk. Endotracheal intubation and mechanical ventilation disrupt respiratory mucosal barriers, increase device-related exposure, and promote biofilm formation (32). Previous studies have shown that mechanical ventilation is associated with acquisition of MDR *A. baumannii*, and that Gram-negative organisms such as *Klebsiella*, *Acinetobacter*, and *P. aeruginosa* are major causes of ventilator-associated pneumonia in very preterm infants (33–37). From a clinical perspective, this finding supports intensified airway infection-prevention measures in infants requiring IMV shortly after admission, including strict aseptic suctioning, optimized ventilator circuit management, and improved oral care (38). Preventive bundles incorporating these components have been shown to reduce ventilator-associated infection rates in neonatal populations (39). In addition, oral care with human milk may enhance mucosal immune defense, while early extubation or transition to noninvasive respiratory support may help reduce biofilm-related infection risk (38, 40). However, IMV in the present model should be interpreted primarily as a marker of early invasive exposure and illness severity rather than as an isolated causal determinant.

A lower 5-minute Apgar score was associated with a higher ESKAPE risk (OR = 0.65 per 1-point increase), likely reflecting impaired perinatal adaptation, hemodynamic instability, and transient immune dysregulation after asphyxia or hypoxia, all of which compromise host defense against opportunistic pathogens (41, 42). Previous studies have similarly identified low Apgar scores as risk indicators for neonatal sepsis. A meta-analysis including 49 studies and 87,548 neonates showed that a 1-minute Apgar score <7 was associated with a significantly increased risk of neonatal sepsis (OR = 7.56, 95% CI: 3.39–11.73) (43). Another systematic review from sub-Saharan Africa reported that a low 5-minute Apgar score was also strongly associated with sepsis risk (OR = 2.55, 95% CI: 1.46–4.45) (44). In addition, studies from NICU settings have suggested that low Apgar scores are associated with multidrug-resistant organism (MDRO)-related neonatal sepsis, with major isolates including ESKAPE pathogens such as *K. pneumoniae*, *A. baumannii*, and *S. aureus* (45, 46). Taken together, these findings suggest that the Apgar score may serve not only as a marker of immediate postnatal adaptation, but also as a practical early indicator of infection vulnerability in high-risk neonatal populations.

Vasoactive exposure within the first 24 h was also identified as an important predictor in the final model (OR = 3.23). This likely reflects the role of early hemodynamic instability as a marker of illness severity and increased susceptibility to hospital-acquired pathogens. Infants requiring vasoactive support are often those with severe respiratory distress syndrome or perinatal asphyxia, both of which have been linked to impaired immune function (47). In addition, these critically ill infants typically require prolonged NICU care and more frequent invasive procedures (e.g., suctioning), which may disrupt anatomical and physical barriers and increase exposure to ESKAPE pathogens (48). Systemic hypoperfusion may further compromise intestinal mucosal integrity, facilitating translocation of pathogens from the gut (48). Catecholamine exposure, whether endogenous or exogenous, may also contribute to immune dysregulation by suppressing proinflammatory responses and promoting anti-inflammatory pathways such as IL-10 production, thereby impairing host defense (49). From a clinical perspective, these findings suggest that infants requiring early vasoactive support may benefit from closer monitoring and intensified infection-prevention strategies, including careful circulatory management, enteral feeding monitoring, and strict adherence to aseptic techniques.

GA at birth was inversely associated with the risk of hospital-acquired ESKAPE colonization or infection in very preterm infants, highlighting the close link between immune maturity and infection susceptibility. Preterm infants have immature host defenses, including reduced barrier integrity and attenuated innate and Th1-polarizing immune responses, which may increase vulnerability to opportunistic hospital-acquired pathogens (50). Meanwhile, delayed establishment of intestinal flora allows ESKAPE pathogens including *K. pneumoniae* to become dominant colonizers in the early postnatal period (51). Several studies have demonstrated an inverse correlation between GA and MDR bacterial infection. A prospective NICU cohort study in Malaysia reported that the intestinal colonization rate of MDR *K. pneumoniae* and *Escherichia coli* reached 52% in preterm infants <37 weeks of gestation, with evident clonal transmission (52). Multiple investigations of *K. pneumoniae* outbreaks also indicated that smaller GA was associated with higher infection risk (53). However, no study to date has quantified the dose-response relationship between GA and ESKAPE infection in very preterm infants ≤32 weeks. For smaller very preterm infants, early routine care after admission should include earlier attention to skin barrier maintenance, more delicate management of oral and intestinal microecology (e.g., promotion of breastfeeding), and reduction of colonization pressure from opportunistic pathogens through enhanced environmental cleaning.

Regarding model development, previous studies have reported prediction tools for neonatal MDRO infection or late-onset sepsis. For example, Zhou et al. developed a model for MDRO infection based on LBW, advanced maternal age, prolonged antibiotic use, and MDRO colonization (AUC = 0.788) (18). Huang et al. established a nomogram for late-onset sepsis using thyroid function, BW, tracheal intubation, and duration of umbilical venous catheterization (AUC = 0.855) (19). Compared with these models, our nomogram has several features that may be clinically relevant. First, the study specifically focused on very preterm infants with GA ≤32 weeks, a subgroup with marked physiological immaturity and high susceptibility to hospital-acquired ESKAPE colonization or infection. Second, all predictors included in the model are routinely available within the first 24 h after birth, making early risk assessment possible without waiting for later laboratory results. Third, the retained predictors are closely related to major NICU care domains, including airway management, circulatory support, and perinatal assessment, which may make the model easier to interpret and apply in bedside practice. Overall, these features may help identify high-risk infants earlier and support more targeted allocation of infection control resources.

The nomogram enables early risk stratification of very preterm infants for hospital-acquired ESKAPE colonization or infection using routine clinical variables available within 24 h after birth. In clinical practice, the online calculator can be used to estimate individualized risk and identify infants who may benefit from intensified infection-control measures. Infants whose predicted risk exceeds the optimal threshold identified in this study (predicted probability ≥0.157; total nomogram score ≥61.2) may be considered high risk and receive enhanced preventive measures, including strict contact isolation, dedicated nursing documentation, invasive procedures performed by senior nurses, frequent environmental cleaning, and proactive microbiological surveillance (54). However, this threshold should be regarded as exploratory and should be externally validated before routine clinical implementation.

Several limitations should be acknowledged. First, this was a single-center retrospective study, and local epidemiology and clinical practice may limit generalizability. Second, although the EPV was acceptable, the relatively limited number of outcome events may still affect model stability. Therefore, external multicenter validation is needed to further confirm the robustness and generalizability of the model. Third, the 10-year study period may have introduced temporal heterogeneity due to changes in infection-control practices, antimicrobial use, and pathogen epidemiology. In addition, because only infants with at least one microbiological culture were included, selection and detection bias cannot be excluded, and the predominance of respiratory specimens may partly reflect local sampling practices. Finally, several potentially important determinants of ESKAPE acquisition and transmission, including microbial genomic data, colonization pressure, and environmental factors, were not incorporated into the model. Despite these limitations, the model provides a practical framework for early risk stratification in very preterm infants. Future studies should focus on multicenter external validation, model refinement using epidemiological and environmental data, and evaluation of whether risk-guided infection control strategies can improve patient outcomes.

## 5 Conclusions

In conclusion, we developed an internally validated nomogram for early prediction of hospital-acquired ESKAPE colonization or infection in very preterm infants using routinely available indicators within 24 h after birth. The model may support early risk stratification and infection-control prioritization in the NICU. Further multicenter prospective validation is required before clinical implementation.

## Data Availability

The raw data supporting the conclusions of this article will be made available by the authors, without undue reservation.
