# Supplementary material for: Development and internal validation of a nomogram for early prediction of hospital-acquired ESKAPE colonization or infection in very preterm infants using indicators available within 24 hours
Source: Front Pediatr. 2026 Jun 8;14:1847533. doi: 10.3389/fped.2026.1847533 (PMC13283798; doi:10.3389/fped.2026.1847533)
Supplement: Supplementary file 1 [file Table1.docx]

Results of Multivariate Logistic regression for Training Cohort

| **Characteristic** | **N** | **Event N** | **OR** | **95% CI** | **p-value** |
| --- | --- | --- | --- | --- | --- |
| Gestational_age | 325 | 53 | 0.97 | 0.94, 1.00 | 0.079 |
| 5_minute_Apgar_score | 325 | 53 | 0.65 | 0.53, 0.82 | <0.001 |
| Initial_invasive_mechanical_ventilation |  |  |  |  |  |
| NO | 176 | 14 | — | — |  |
| YES | 149 | 39 | 1.91 | 0.89, 4.09 | 0.097 |
| Vasoactive_exposure_within_first_24h |  |  |  |  |  |
| NO | 228 | 22 | — | — |  |
| YES | 97 | 31 | 3.23 | 1.66, 6.30 | <0.001 |
| Abbreviations: CI = Confidence Interval, OR = Odds Ratio | | | | | |
