# Supplementary figures and images for: Development and internal validation of a nomogram for early prediction of hospital-acquired ESKAPE colonization or infection in very preterm infants using indicators available within 24 hours
Source: Front Pediatr. 2026 Jun 8;14:1847533. doi: 10.3389/fped.2026.1847533 (PMC13283798; doi:10.3389/fped.2026.1847533)

# ROC Curve for ESKAPE Prediction

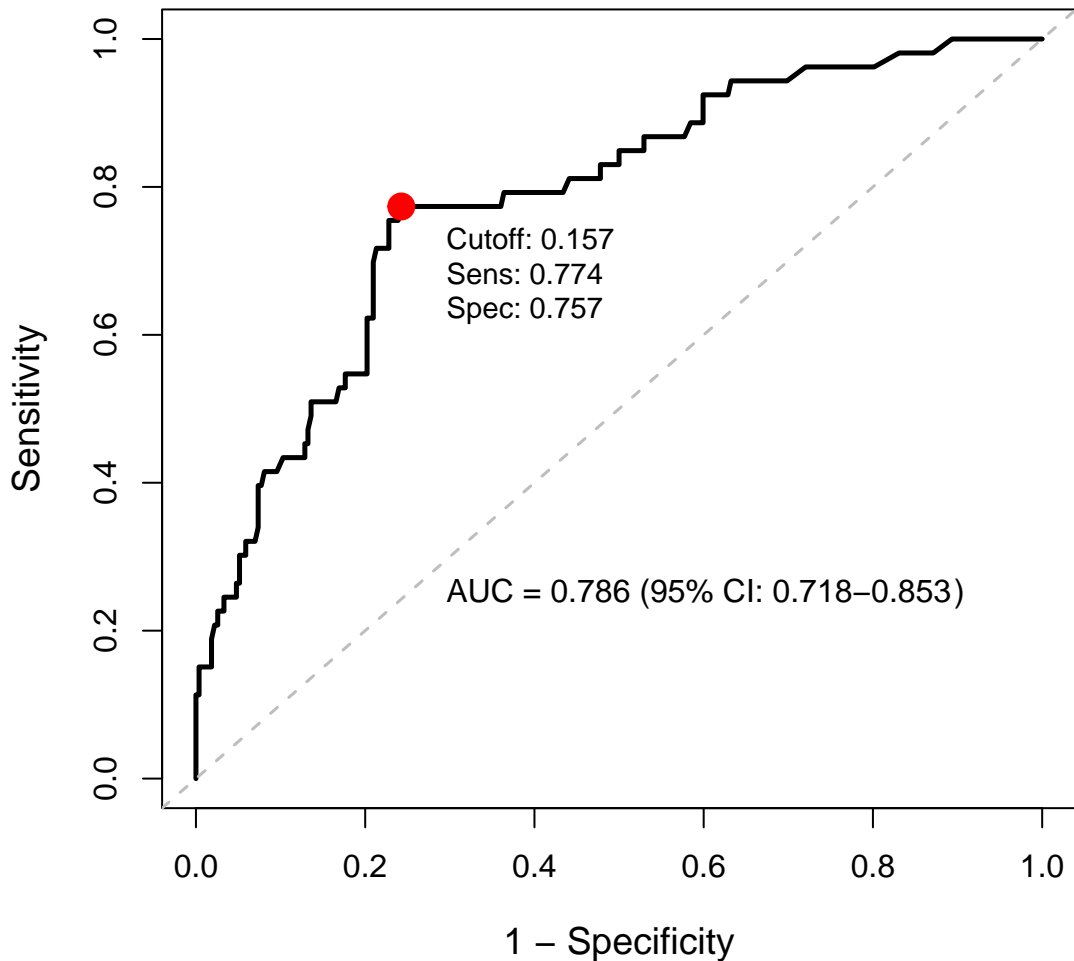

Supplement: Supplementary file 2 [file Image1.pdf]
